# Supplementary material for: Comparative analysis of data-driven models for spatially resolved thermometry using emission spectroscopy
Source: PLoS One. 2025 Jan 24;20(1):e0317703. doi: 10.1371/journal.pone.0317703 (PMC11760626; doi:10.1371/journal.pone.0317703)
Supplement: Attachment — Submitted filename: PONE-D-24-35923-Response to the Reviewers Comments.pdf [file pone.0317703.s001.pdf]

Comments to the Author

We would like to sincerely thank all the reviewers for their insightful comments and constructive suggestions. Your thorough evaluation and thoughtful feedback have greatly contributed to improving the clarity, depth, and overall quality of the submitted manuscript. We truly appreciate the time and effort you have dedicated in reviewing our work, and your valuable input has been instrumental in refining our research. Thank you once again for your contributions to this process.

Reviewer #1:

1.1 Some Keywords are not refined enough (e.g The human body posture estimation)

Response: Thank you for your valuable comment. We appreciate your suggestion regarding refining the keywords. We propose using "Spatially Resolved Thermometry, Feature Engineering, and Machine Learning" as more precise keywords to better reflect the focus of our study. However, we would like to note that PLOS ONE does not provide an option to set keywords directly. We hope this clarification helps and thank you again for your insight.

1.2 The introduction can be enriched by briefly describe the state-of-the-art in the title of this study and provide more recently related references to support foundation of this studies to better context for the current research. I will recommend citing recently published related papers;
a. Internet of Medical Things (IoMT): Applications, Challenges, and Prospects in a Data-Driven Technology. In: Chakraborty, C., Khosravi, M.R. (eds) Intelligent Healthcare. pp 299–319, Springer, Singapore. https://doi.org/10.1007/978-981-16-8150-9_14
b. Statistical Analysis of Stakeholders Perception on Adoption of AI/ML in Sustainable Agricultural Practices in Rural Development. In X. S. Yang, S. Sherratt, N. Dey, & A. (. Joshi (Ed.), Proceedings of Ninth International Congress on Information and Communication Technology. ICICT 2024 2024. Lecture Notes in Networks and Systems. 1003. Springer, Singapore. doi:https://doi.org/10.1007/978-981-97-3302-6_11

Response: Thank you for your valuable suggestion. The recommended references were included and cited in the resubmitted manuscript.

1.3 The problem that author is trying to address in this paper is not clear yet, (clearer motivation for study is required)

Response: We apologize for the lack of clarity in terms of the problem. To address this comment, we have further modified the motivation part:

"In this work, we systematically address the challenge of recovering spatial temperature information from line-of-sight emission spectra measurements using data-driven modelling approaches. We first explore multiple feature engineering methodologies, and combine them with various machine learning (ML) algorithms. In parallel, we explore end-to-end deep learning (DL) algorithms for temperature distribution reconstruction purposes." is revised to *"The primary objective of this study is to develop and evaluate a comprehensive framework for spatially resolved temperature estimation from emission spectra, integrating feature engineering and ML approaches. By addressing the limitations of conventional Deep Learning (DL) and exploring the potential of hybrid methodologies, this research aims to enhance predictive accuracy and reliability, ultimately contributing to advancements in various applications ranging from industrial combustion monitoring to environmental assessments."*

1.4 Add the contribution of this study and study organization towards end of Introduction Section

Response: We would like to thank the reviewer for their comment. A dedicated paragraph which summarizes the key contributions of the study, and highlights the innovative aspects of the research has been included, i.e.,

"The contributions of the work are as follows:

1.To the best of the authors' knowledge, this is the first work which systematically explores and compares the use of feature engineering and machine learning vs deep learning algorithms for spatially resolved temperature measurement estimation.

2. The top performing method involves a combination of signal representation-based feature extraction, and blending-based ensemble machine learning, achieving cutting-edge performance, which outperforms state-of-the-art approaches and deep learning algorithms.

3. It is demonstrated that the proposed approach is capable of accurately providing spatially resolved temperature estimates using lower resolution ES spectra, compared to published work, which offers an important cost advantage for real-world applications."

Moreover, an outline of the manuscript structure was included at the end of the introduction:

"The manuscript is organized as follows: Section 2 provides a detailed overview of the related works and the state-of-the-art methodologies in emission spectroscopy and temperature estimation. Section 3 outlines the methodology, including the data collection process, feature extraction techniques, and the machine learning models employed. In Section 4, the results of the experiments are presented, followed by a discussion of the findings in Section 5. Finally, Section 6 concludes the study and outlines future research directions."

1.5 In 4-5- lines, authors should summarize the literature gaps identified before starting the methodology (Kindly relate your work in the context of existing work)

Response: We appreciate the reviewer's comment. To address this, we have further modified the related work section and include a table to summarize and compare all these methods. Moreover, as suggested by the reviewer, we have included a paragraph summarizing the gaps in the existing literature on temperature estimation from emission spectroscopy:

"Despite various advancements in both optimization-based and machine learning methods for temperature estimation from emission spectroscopy, significant gaps remain. Optimization-based approaches are often highly sensitive to initial conditions and hyperparameters, limiting their robustness in complex scenarios. Machine learning models, while promising, have primarily focused on simple temperature distributions and have struggled to generalize to more complex profiles due to insufficient feature engineering. Furthermore, few studies have explored the role of advanced feature extraction techniques to enhance model performance, especially in handling non-uniform temperature fields. This study addresses these gaps by incorporating comprehensive feature engineering with machine learning to improve accuracy in complex temperature estimations."

This streamlined version clarifies the identified literature gaps and emphasizes the study's contribution.

1.6 Author should re-check the correctness of equations especially equations 8 and 14

Response: The equations have been re-checked, thank you.

1.7 Services of language expert are required

Response: The manuscript underwent review by a native English speaker and language issues were rectified.

1.8 Image quality needs to be improved

Response: All figures were reproduced at higher resolution (300 DPI) and adjusted labels and legends for better visibility.

1.9 Ensure that all Figures are properly labeled and referenced

Response: Verified and corrected figure labels and cross-referenced all figures in the text.

1.10 Authors should include the future work of this study

Response: Thank you for your valuable suggestions. In response, we have included a future work section at the end of the manuscript. The future directions for this study are as follows:

"*As for future work, there are several aspects could be pursued.*

1. Data generation. More diverse temperature profile datasets could be generated, so as to further expand the application scenarios and assess the limitations of the proposed method.

2. Feature engineering and learning. More detailed hyper-parameter search, such as window size selection, and feature engineering methods, such as other dimensionality reduction methods, could be explored, for better performance. In addition to current feature engineering technologies, advanced feature learning technologies such as contrastive learning and self-supervised learning could be further explored, to extract more effective features for better performance.

3. Incorporating hybrid machine learning approaches, where feature engineering is used to augment deep learning models, has the potential to significantly enhance prediction accuracy for temperature estimation from emission spectra. Hybrid methodologies capitalize on the strengths of both classical feature extraction techniques and modern deep learning architectures. By integrating domain knowledge through feature engineering with the learning capabilities of deep learning models, it is possible to create more robust and accurate predictive models."

Reviewer #2:

2.1 The title is clear and concise. However, consider specifying in the title that you compare different data-driven models, as this may better capture the comprehensive scope of your research.

Response: Thank you for your valuable comment, we have revised the title to: *"Comparative Analysis of Data-Driven Models for Spatially Resolved Thermometry Using Emission Spectroscopy"*.

2.2 Your systematic exploration of data-driven models is well-structured. Ensure that the methodology section provides detailed explanations of the implementation of feature engineering techniques, including physics-guided transformations, polynomial approximation-based features, and PCA.

Response: Thank you for your kind suggestion, we checked the details of the descriptions of the feature engineering techniques employed in the study, and ensured that each technique is explained in the context of its relevance to the study.

2.3 The methodology, results, and conclusions are clearly presented. Consider adding more discussion on practical implications and potential applications of your findings.

Response: To address this comment, we expanded the discussion as recommended by the reviewer, as follows:

"The findings of this study can contribute to the advancement of both measurement accuracy and the practical application of these techniques in various industries and research domains.

1. Advancements in Temperature Estimation Accuracy: The high performance of the feature-engineered models, demonstrated by the low RMSE and high correlation coefficients, suggests that data-driven approaches can enhance the accuracy of temperature measurements in scenarios where conventional methods may struggle. For instance, in spatially resolved thermometry, where detailed temperature profiles are required, the ability to accurately capture temperature variations across a region can lead to better control in industrial processes such as combustion monitoring, materials processing, and chemical reactors. Improved accuracy can lead to better operational efficiency, energy savings, and enhanced safety, particularly in environments where precise temperature control is critical.

2. Applications in Industrial and Environmental Monitoring: The findings from this study can be directly applied to improve real-time monitoring systems in various industrial applications. In industrial combustion, for example, accurate temperature monitoring enables operators to optimize fuel consumption, reduce emissions, and improve overall process efficiency. The high accuracy of the models in estimating temperatures from emission spectra can also facilitate the development of more advanced, automated monitoring systems for factories, power plants, and industrial furnaces, reducing the need for manual interventions.

3. Technological Improvements in Emission Spectroscopy Systems: The integration of the proposed models into existing emission spectroscopy systems could lead to technological improvements in terms of spatial resolution and measurement efficiency. Current systems may benefit from incorporating machine learning algorithms capable of analyzing spectral data more efficiently and with greater accuracy. This could pave the way for next-generation spectroscopic equipment that not only measures emission spectra with high spatial resolution but also provides real-time temperature data with minimal manual intervention. More importantly, it does not affect current hardware design, but empower the spatial measurement capability with intelligent algorithm embedding. These advancements could be

particularly beneficial in scenarios requiring continuous monitoring and spatial resolution such as manufacturing processes, environmental assessments, and energy production."

2.4 It is important that the description of the CNN models includes details on their architecture, training process, and evaluation metrics to provide a complete understanding of their performance relative to classical methods.

Thank you very much for your valuable comment, as for the CNN models, we use the default settings of these models. To address this, we included references for interested readers to obtain further details in regards to their architectures. In addition to this, we provided additional details about the training process and described the evaluation metrics for the readers' convenience as follows:

"During training, the Adam optimizer [47] was used, and a weight decay of 1e−4 was selected to alleviate overfitting. The learning rate was preliminarily determined by the method suggested in [48], however, it was further decreased as the selected deep learning models were prone to overfitting the training set, particularly, after removing the dropout and normalization operations, required for the regression application. Thus, the learning rate was varied and determined for the specific CNN architecture. In order to better navigate the training direction, the warm-up operation [49] was used to train the networks in the first ten epochs, during which, the learning rate was gradually increased from a tiny value to the selected learning rate. Such a soft activation of training supports the appropriate choice of training direction. During the normal training process, the cosine annealing learning rate schedule [49] was used, which helps to avoid falling into local minima."

2.5 The presentation of performance metrics (RMSE: 64.3, RE: 0.017, RRMSE: 0.025, R: 0.994) is effective in highlighting the superiority of the light blender model. Ensure that the implications of these metrics are discussed in the context of practical applications

Response: Thank you for your insightful comment. In response, we have added a section in the conclusion to discuss the practical implications of the performance metrics and their relevance to real-world applications, as shown in the response for comment 2.3.

2.6 The acknowledgment that end-to-end CNNs are competitive but not superior is crucial. Consider providing more details on the specific limitations of CNNs in this context.

Response: We expanded the CNN section of the Discussion. Here, we focused on articulating the limitations of CNNs compared to other methods used in the study, emphasizing why they may not always be the best choice for temperature estimation from emission spectra. The following paragraphs have been included:

"Despite the well-documented success of Convolutional Neural Networks in various data-driven applications such as image classification, object detection, and time-series forecasting, their performance in the context of spatially resolved temperature measurement using emission spectroscopy was found to be inferior to classical machine learning methods in this study. Several key reasons contribute to this outcome:

- *Loss of Spectral Characteristics During Convolution: Spectral data is characterized by its peaks and troughs, which directly correlate with the physical properties of the gas mixture, such as concentration and temperature distribution. Convolutional filters, designed for translation invariance in images, do not inherently capture these spectral characteristics effectively. As a*

result, important spectral features such as peak intensities and their precise locations might be averaged out during convolution, leading to a loss of critical information necessary for accurate temperature estimation.

- *Challenges with High-Dimensional Spectral Inputs: The high-dimensional nature of the spectral data (e.g., 6799 emission lines) poses a challenge for CNNs, which rely on down-sampling through pooling layers to reduce computational complexity. However, this reduction can lead to information loss, especially when the input spectra contain fine-grained details that are crucial for distinguishing subtle variations in temperature. In contrast, feature engineering methods like polynomial approximations and PCA preserve this high-dimensional information more effectively by focusing on extracting relevant features without losing the global context of the spectral data.*
- *Insufficient Feature Representation Learning: CNNs tend to learn hierarchical features through multiple layers of convolutional filters, which is highly beneficial for image data. However, in the case of emission spectra, where the relevant features are not hierarchical in nature, the learned representations may not align with the true underlying patterns. As demonstrated in this study, feature engineering techniques such as physics-guided transformations and signal representation features provide a more direct representation of the spectral data, allowing classical machine learning models to leverage these features more effectively.*
- *Feature Engineering in Domain-Specific Knowledge: The feature engineering techniques employed in this study incorporate domain-specific knowledge through physics-based transformations and signal representation features. These features are explicitly designed to capture the relationships between temperature, concentration, and spectral intensities. This contrasts with CNNs, which lack such domain-specific priors and rely entirely on the data to learn these relationships. As a result, CNNs often require larger datasets and extensive training to match the performance of models utilizing engineered features."*

2.7 (This is addressed in 2.4) Expand on the architecture of the CNN models used in the study. Describe the layers, activation functions, and any preprocessing steps involved. Also, include details on the training process, such as hyperparameters and training duration.

Response: Thank you very much for your comment, this has been addressed in 2.4

2.8 Offer a more in-depth comparative analysis between feature engineering methods and end-to-end CNNs. Include a discussion on why feature engineering methods outperform CNNs in this context and any potential reasons for CNNs' lower performance.

Response: Thank you for your insightful comment. The reasons behind the better performance of feature engineering and ML vs CNN have been addressed in the response to comment 2.6.

2.9 Provide a detailed analysis of the performance metrics (RMSE, RE, RRMSE, and R) and explain their significance. Discuss how these metrics translate to practical advantages or limitations in temperature measurement.

Response: Thank you for this comment. The following paragraphs were included in Section of Machine Learning Model Performance:

"The evaluation of the proposed models was conducted using four key performance metrics: Root Mean Square Error (RMSE), Relative Error (RE), Relative Root Mean Square Error (RRMSE), and the

correlation coefficient (R). Each metric provides unique insights into the accuracy and reliability of the temperature estimations from emission spectra. Below is a detailed analysis of these metrics and their significance:

Root Mean Square Error (RMSE)

This measures the square root of the average squared differences between the predicted and actual temperature values. RMSE provides a clear indication of the model's overall predictive accuracy. A lower RMSE indicates that the model's predictions are closer to the actual temperature values, which is crucial for applications requiring precise temperature control. In practical temperature measurement scenarios, such as combustion monitoring and industrial process control, maintaining a low RMSE is essential to avoid operational inefficiencies or safety issues. For example, a high RMSE in a combustion process might result in suboptimal fuel usage or increased emissions, leading to higher costs and environmental impact.

Relative Error (RE)

Relative Error quantifies the absolute difference between the predicted and actual temperatures, expressed as a fraction of the actual temperature value. This provides insight into the accuracy of predictions relative to the magnitude of the actual values. It is particularly useful for comparing the model's performance across different temperature ranges. A low RE value suggests that the model's predictions are consistently accurate across varying temperature levels. This is advantageous in applications such as environmental monitoring, where temperature variations may span a wide range, and consistent accuracy is required to make reliable assessments.

Relative Root Mean Square Error (RRMSE)

RRMSE is a normalized version of RMSE that expresses the error as a fraction of the average of the actual values and is useful for comparing the model's performance across different datasets or scenarios with varying temperature scales. A low RRMSE value indicates that the model performs well relative to the typical values of the temperature data. This is beneficial for assessing the model's robustness across different experimental conditions or for generalizing to new datasets that may have different temperature profiles.

Correlation Coefficient (R)

The correlation coefficient, R, measures the strength and direction of the linear relationship between the predicted and actual temperatures. An R value close to 1 indicates a strong positive linear relationship, meaning that the model captures the overall trend of temperature variations effectively. A high R value is crucial for applications where understanding temperature trends and patterns is more important than absolute accuracy. For example, in climate modeling or atmospheric studies, capturing the general trend of temperature changes can be more informative for decision-making than minimizing individual prediction errors.

The combination of these metrics provides a comprehensive evaluation of the model's performance. For instance, a low RMSE and RE indicate high accuracy, making the model suitable for precise temperature measurements in industrial processes. A low RRMSE shows that the model's performance is consistent relative to the overall magnitude of temperature values, which is advantageous in varying experimental conditions. Finally, a high R value confirms that the model effectively captures the temperature trends, making it valuable for applications such as environmental assessments and research in temperature dynamics."

2.10 Elaborate on the practical implications of the results. How do they impact the field of spatially resolved thermometry and emission spectroscopy? Discuss any potential applications or improvements in technology that could result from your findings.

Response: To fully address the reviewer's comment we expanded the Discussion and Conclusion sections to how the findings contribute to the advancement of these fields, addressed under comment 2.3.

2.11 Include an analysis of potential sources of error in your measurements and predictions. Discuss how these errors could affect the reliability of your method and any steps taken to mitigate them.

Response: Thank you for arising this valuable comment. We include a section on error sources as follows:

"Although models work well in the study, we also analyzed the potential error sources that could limit its performance in practice:

1. Model Uncertainty: Model uncertainty refers to the variability in predictions due to the inherent limitations of the model architecture, parameter choices, and the training process. This type of uncertainty is also known as aleatoric uncertainty, which arises from the stochastic nature of the learning algorithm, such as the use of random initial weights in neural networks or random splits in decision trees. In the context of emission spectroscopy, model uncertainty is often introduced by suboptimal model configurations, inappropriate model complexity (e.g., underfitting or overfitting), and the inability of the model to capture the complex non-linear relationships between spectral features and temperature distributions.

2. Data Uncertainty: Data uncertainty, also referred to as measurement or observational uncertainty, is caused by the inherent noise present in the emission spectra due to factors such as instrumentation limitations, fluctuations in the light source, and environmental conditions during data acquisition. The level of data uncertainty is directly influenced by the spectral resolution, the signal-to-noise ratio (SNR), and the quality of the measurements. In emission spectroscopy, noise can manifest as random variations in intensity values, baseline shifts, or the presence of spurious peaks that do not correspond to actual physical phenomena.

3. Epistemic Uncertainty: Epistemic uncertainty arises from the lack of knowledge or information about the underlying data distribution and the limitations in the training data. This type of uncertainty, also known as systematic or structural uncertainty, is present when the model encounters scenarios or data points that were not represented in the training set. In the context of temperature estimation from emission spectra, epistemic uncertainty may manifest as the model's inability to generalize to unseen temperature profiles, gas compositions, or spectral regions with unusual characteristics. It typically occurs when there are insufficient training samples to cover the entire range of possible temperature distributions or when there are gaps in the data that prevent the model from learning the full data manifold. Epistemic uncertainty can lead to poor generalization and erroneous predictions when the model is exposed to out-of-distribution (OOD) data. "

2.12 Provide a detailed description of the datasets used, including their size, diversity, and any preprocessing steps. This will help readers understand the context and limitations of the data. The detailed description of the dataset is as follows:

Response: In addition to the original dataset description, we added a detailed dataset description in the section of data generation, i.e.,

" The detailed description of the dataset is as follows:

1. Size and Composition: The primary dataset used for this study consists of a total of 28000 labeled emission spectra, each corresponding to a unique temperature profile. The spectra were generated using profiles of variants of the dual-peak Gaussian functions to ensure a diverse representation of temperature conditions. The dataset was divided into three subsets: training, validation, and test sets with the ratio of 70%, 15%, and 15%.

2. Diversity of Temperature Profiles: The dataset includes a wide range of temperature profiles along with a wide range of mole fraction profiles. This diversity was introduced to mimic real-world scenarios, such as non-homogeneous temperature fields in industrial furnaces or environmental monitoring settings. The temperature values in the dataset range from 1100 K to 3400 K, covering diverse temperature scenarios.

3. Spectral Resolution and Variability: The spectral data were generated with a low resolution of 4 cm^{-1}, which was subsequently blurred by instrument noises, to simulate the actual scenario, and also increase the difficulties in temperature measurement. The low-resolution spectra were resampled every 0.1 cm-1 resulting in 6,799 intensity values per spectrum. "

2.13 I recommend conducting additional experiments to test your proposed method in varied conditions or with different types of emission spectra to further validate its robustness.

Response: Thank you for your thoughtful suggestion. We agree that testing the proposed method under varied conditions is essential for validating its robustness, particularly in engineering applications. As you noted and mentioned in comment 2.11, robustness plays a crucial role, and this is addressed by introducing complexity in the dataset generation process to simulate a wide range of combustion scenarios involving emissions such as CO, CO_2, and water under a large variety of temperature ranges and profiles.

The current research focuses specifically on combustion scenarios, and by incorporating varied profiles and spectral configurations, we aimed at capturing the complexity inherent in these processes. The results demonstrate that while state-of-the-art methods struggle with these complex cases, the proposed approach effectively handles them. This strong performance on the test set reinforces the robustness of the proposed method.

Although we did not explicitly test for generalization to out-of-distribution (OOD) data, we have discussed the potential errors associated with OOD scenarios. We also acknowledge the importance of further validation and, as stated in the conclusion, *"Datasets of diverse conditions could be generated, so as to further expand the application scenarios and assess the limitations of the proposed method*." This future work will allow us to explore additional scenarios and strengthen the robustness of the approach across a broader range of conditions.

2.14 Your conclusion appropriately emphasizes the effectiveness of the feature engineering methodology. Ensure that the rationale for why these methods are superior is clearly articulated.

Response: Thank you for your valuable comments, and this has been addressed in comment 2.6.

2.15 The conclusion effectively communicates the strong performance of the light blender model. It might be helpful to elaborate on how these results impact the field of spatially resolved thermometry.

Response: Thank you very much for your valuable suggestion. We include a further paragraph, discussing this in the conclusion section on how it will impact the related fields,

"Technological Improvements in Emission Spectroscopy Systems: The integration of the proposed models into existing emission spectroscopy systems could lead to technological improvements in terms of spatial resolution and measure efficiency. Current systems may benefit from incorporating machine learning algorithms capable of analyzing spectral data more efficiently and with greater accuracy. This could pave the way for next-generation spectroscopic equipment that not only measures emission spectra with spatial resolution but also provides real-time temperature data with minimal manual intervention. More importantly, it does not affect current hardware design, but empower the spatial measurement capability with intelligent algorithm embedding. These advancements could be particularly beneficial in scenarios requiring continuous monitoring and spatial resolution such as manufacturing processes, environmental assessments, and energy production."

Reviewer #3:

In this paper, the authors have conducted a study on the enhancement of spatially resolved thermometry using line-of-sight emission spectroscopy by applying data-driven models to improve temperature measurement accuracy in non-homogeneous fields. While the study addresses a challenge in emission spectroscopy, several suggestions are provided to improve the clarity and depth of the methodology before recommending it for publication:

3.1 The authors should look into hybrid machine learning approaches, where feature engineering is used to augment deep learning models? This could potentially offer further improvements in prediction accuracy.

Response: Thank you for your insightful comments and suggestions regarding the exploration of hybrid machine learning approaches in the manuscript. We appreciate your emphasis on the potential benefits of integrating feature engineering with deep learning models to enhance prediction accuracy. While the current work primarily focuses on evaluating the performance of classical machine learning models and deep learning architectures independently, we recognize that hybrid approaches can indeed leverage the strengths of both methodologies. The combination of feature engineering and deep learning has shown promise in various applications, particularly in handling complex data types such as emission spectra. In light of your suggestion, we have added the following paragraph as part of future work in the conclusion part as follows:

"Incorporating hybrid machine learning approaches, where feature engineering is used to augment deep learning models, has the potential to significantly enhance prediction accuracy for temperature estimation from emission spectra. Hybrid methodologies capitalize on the strengths of both classical feature extraction techniques and modern deep learning architectures. By integrating domain knowledge through feature engineering with the learning capabilities of deep learning models, it is possible to create more robust and accurate predictive models."

3.2 Please provide a more detailed explanation or justification of the assumption on thermal equilibrium and the use of dual-peak Gaussian functions for temperature profiles, particularly in scenarios where these conditions might not hold true, for example, turbulent flows.

Response: Thank you for your valuable comment. The assumption of thermal equilibrium is commonly employed in spectroscopy-based temperature measurements, where it is assumed that energy exchange between molecules occurs rapidly enough to establish a stable temperature distribution along the light path. This simplification holds particularly well in controlled environments with minimal temperature fluctuations and homogeneous gas composition. In combustion scenarios, the assumption is reasonable because the rapid reaction zone, where the fuel and oxidizer interact, tends to exhibit the highest temperatures, while temperatures decrease gradually within the fuel flow and outside the oxidizer due to heat transfer. This creates a temperature gradient that can be modeled using dual-peak Gaussian functions, as has been demonstrated in previous research [16]. Dual-peak Gaussian profiles have been successfully used in data generation and experimental validation to approximate such combustion conditions.

In the current study, fluctuations to the dual-peak Gaussian profiles were introduced, as shown in Figure 3(b), to account for more complex and realistic scenarios. This additional variability ensures that the proposed model is not overly reliant on a specific profile shape, thereby enhancing its generalizability. By incorporating these fluctuations, the generated profiles also encompass situations where the temperature distribution may deviate from a perfect dual-peak Gaussian shape, such as in turbulent

flows. Consequently, the proposed model performs well not only in idealized cases but also in more complex, non-equilibrium conditions.

3.3 The Related Works sections consist of very little information, therefore, I would recommend the authors to incorporate more information regarding previous research and their outcomes in this specific area of using data-driven models for temperature measurement. Additionally, adding a table as well to highlight the previous research done in this field and how it compares to this research.

Response: Thank you for your comment, we modified the related work section to accommodate more information, and provide a comparison between the SOTA works, including a summary Table, as follows:

"The application of data-driven models in emission spectroscopy for spatially resolved temperature measurement has been explored in several studies, each offering distinct contributions but also facing inherent challenges. The primary research efforts in this domain can be categorized into optimization-based approaches and machine learning-based methods:

1. ***Optimization-Based Approaches:*** *Early works by Kim and Song [3] and Ren et al. [5] utilized regularization strategies and optimization algorithms to extract spatially resolved temperature information from line-of-sight spectra. These methods leveraged forward simulation models to iteratively optimize the fit between simulated and actual spectra. Kim et al. [3] proposed a fast inversion scheme for spatial temperature measurement in large-scale furnaces, relying on spectral remote sensing (SRS) technology. Ren et al. [5] combined Levenberg-Marquardt optimization and Tikhonov regularization to recover temperature profiles of* CO_2*. While these methods can provide high accuracy under ideal conditions, their performance is highly sensitive to the initial guesses of temperature distributions and the selection of hyperparameters, making them less robust for complex scenarios.*
2. ***Machine Learning-Based Approaches:*** *Recent advancements have shifted the focus towards machine learning (ML) techniques, which offer the advantage of learning from data without requiring explicit physics-based models. Studies by Cięszczyk et al. [14] and Ren et al. [15] utilized multilayer perceptron (MLP) networks to estimate spatial temperature distributions. Cięszczyk et al. [14] developed a model based on the ratios of radiation intensities at multiple* CO_2 *lines and applied it to reconstruct plume temperature distributions. Ren et al. [15] explored a more complex MLP architecture with three hidden layers and applied it to retrieve temperature and concentration profiles from infrared emission spectra. While effective for relatively simple temperature distributions, these models struggled to generalize to more complex and non-uniform profiles due to their reliance on raw spectral data without feature engineering.*
3. ***Comparative Analysis of Feature Engineering:*** *Few studies have systematically explored the role of feature engineering in enhancing model performance for spatial temperature measurement. The work by Wei et al. [12] introduced deep neural networks (DNNs) combined with feature extraction techniques to estimate temperature distributions in methane flames. However, their feature extraction was limited to standard statistical descriptors, which did not fully exploit the underlying structure of the spectral data. In contrast, the current work introduces a comprehensive feature engineering methodology, including signal representation and physics-guided transformations, which significantly improves the model's ability to handle complex temperature profiles.*

Table 1 provides a comparison of previous research efforts in this area, summarizing their methodologies, datasets, models used, and key findings:

Study	Methodology	Dataset	Models	Findings	Disadvantages
Kim & Song (2005) [5]	Regularization and optimization-based inverse method	Simulated furnace spectra	Tikhonov regularization, SRS inversion	Achieved high accuracy in large-scale furnace temperature profiles.	Sensitive to initial guesses and hyperparameters.
Ren et al. (2014) [6]	Levenberg-Marquardt optimization with regularization	CO_2 spectral data	Levenberg-Marquardt optimization	Effective for uniform temperature profiles.	Limited scalability to complex, non-uniform profiles.
Cięszczyk et al. (2015) [16]	Ratio of radiation intensities and MLP	CO_2 line intensities	MLP (single hidden layer)	Good performance for uniform plume temperature estimation.	Struggled with complex and non-Gaussian temperature distributions.
Ren et al. (2019) [16]	Infrared emission spectra and MLP with three hidden layers	Simulated temperature profiles	MLP (three hidden layers)	Demonstrated feasibility of MLP for spatial temperature reconstruction.	Required substantial tuning and feature extraction was minimal.
Wei et al. (2020) [13]	Deep neural networks with basic feature extraction	Methane flame spectra	Deep neural networks	Achieved good accuracy for simple profiles; first to apply feature engineering in ES.	Limited feature set and lacked robustness for complex scenarios.

"

3.4 I would suggest the authors to include a section focusing on uncertainty quantification and how the model's confidence intervals or error margins behave under various conditions.

Response: Thank you for raising this valuable suggestion. We added a section on error sources, as follows:

"Although models work well in the study, we also analyzed the potential error sources that could limit their performance in practice:

1. Model Uncertainty: Model uncertainty refers to the variability in predictions due to the inherent limitations of the model architecture, parameter choices, and the training process. This type of uncertainty is also known as aleatoric uncertainty, which arises from the stochastic nature of the learning algorithm, such as the use of random initial weights in neural networks or random splits in decision trees. In the context of emission spectroscopy, model uncertainty is often introduced by suboptimal model configurations, inappropriate model complexity (e.g., underfitting or overfitting), and the inability of the model to capture the complex non-linear relationships between spectral features and temperature distributions.

2. Data Uncertainty: Data uncertainty, also referred to as measurement or observational uncertainty, is caused by the inherent noise present in the emission spectra due to factors such as instrumentation

limitations, fluctuations in the light source, and environmental conditions during data acquisition. The level of data uncertainty is directly influenced by the spectral resolution, the signal-to-noise ratio (SNR), and the quality of the measurements. In emission spectroscopy, noise can manifest as random variations in intensity values, baseline shifts, or the presence of spurious peaks that do not correspond to actual physical phenomena.

3. Epistemic Uncertainty: Epistemic uncertainty arises from the lack of knowledge or information about the underlying data distribution and the limitations in the training data. This type of uncertainty, also known as systematic or structural uncertainty, is present when the model encounters scenarios or data points that were not represented in the training set. In the context of temperature estimation from emission spectra, epistemic uncertainty may manifest as the model's inability to generalize to unseen temperature profiles, gas compositions, or spectral regions with unusual characteristics. It typically occurs when there are insufficient training samples to cover the entire range of possible temperature distributions or when there are gaps in the data that prevent the model from learning the full data manifold. Epistemic uncertainty can lead to poor generalization and erroneous predictions when the model is exposed to out-of-distribution (OOD) data. "

Moreover, under future work, we recommend considering more complex scenarios for data generation: *"As for future work, there are several aspects could be pursued:*

1. Data generation. Datasets of diverse conditions could be generated,so as to further expand the application scenarios and assess the limitations of the proposed method."

In addition, we would like to mention that the dataset generation process cover a variety of scenarios, so that the model can work well. Therefore, the promising model performance on the test set indicates the effectiveness of the pursued methodology and its superiority compared to other studies. As for the cases that are outside of dataset distribution, i.e., generalization performance, this topic is not addressed in the present research, although the instance of potential errors introduced by OOD data has been discussed.

3.5 I would highly suggest that the section headings are presented in a numerical enumeration to keep a consistently organized flow for other researchers to follow.

Response: Thank you very much for your suggestion.,We carefully revised the paper accordingly by adding numerical enumerations for section headings.

3.6 I would recommend the authors to include additional benchmark algorithms or techniques other than just feature engineering and classical machine learning algorithms, and end-to-end convolutional neural networks to provide a more in-depth comparison on both qualitative and quantitative metrics.

Response: Thank you for your valuable suggestion. As highlighted in the introduction and related work, methodologies other than learning-based approaches often struggle to estimate complex temperature profiles due to the ill-posed nature and high degree of non-linearity of the inverse problem in emission spectroscopy. Classical optimization or physics-based techniques are generally insufficient for such scenarios, which is why the present study focuses on learning-based methodologies.

To the best of our knowledge, this study represents the first systematic exploration of multiple learning-based methods for temperature estimation from emission spectra. We provided a comprehensive comparison with the related state-of-the-art (SOTA) algorithms, demonstrating the performance of various models, including feature engineering-based classical machine learning algorithms and end-to-end convolutional neural networks (CNNs). The superior performance of the final model, as evidenced

by both qualitative and quantitative metrics, supports its effectiveness in addressing this challenging problem.

While we acknowledge the reviewer's suggestion to include additional benchmark algorithms, the focus on learning-based methods is driven by the specific requirements and challenges posed by this complex inverse problem. Nevertheless, future work could explore incorporating hybrid methods or advanced model architectures to further expand the comparison across a broader range of techniques, which has been addressed as potential future research:

"Feature engineering and learning. More detailed hyper-parameter search, such as window size selection, and feature engineering methods, such as other dimensionality reduction methods, could be explored, for better performance. In addition to current feature engineering technologies, advanced feature learning technologies such as contrastive learning and self-supervised learning could be further explored, to extract more effective features for better performance.

Incorporating hybrid machine learning approaches, where feature engineering is used to augment deep learning models, has the potential to significantly enhance prediction accuracy for temperature estimation from emission spectra. Hybrid methodologies capitalize on the strengths of both classical feature extraction techniques and modern deep learning architectures. By integrating domain knowledge through feature engineering with the learning capabilities of deep learning models, it is possible to create more robust and accurate predictive models."

3.7 I would suggest the author to provide a more comprehensive explanation of why CNNs underperformed, especially given their success in many data-driven applications. This can add depth to the discussion.

Response: Thank you for your valuable comment. We added the following analysis in the manuscript to answer why feature engineering methodologies are better than CNN in the specific application area:

"Despite the well-documented success of Convolutional Neural Networks in various data-driven applications such as image classification, object detection, and time-series forecasting, their performance in the context of spatially resolved temperature measurement using emission spectroscopy was found to be inferior to classical machine learning methods in this study. Several key reasons contribute to this outcome:

Loss of Spectral Characteristics During Convolution: Spectral data is characterized by its peaks and troughs, which directly correlate with the physical properties of the gas mixture, such as concentration and temperature distribution. Convolutional filters, designed for translation invariance in images, do not inherently capture these spectral characteristics effectively. As a result, important spectral features such as peak intensities and their precise locations might be averaged out during convolution, leading to a loss of critical information necessary for accurate temperature estimation.

Challenges with High-Dimensional Spectral Inputs: The high-dimensional nature of the spectral data (e.g., 6799 emission lines) poses a challenge for CNNs, which rely on down-sampling through pooling layers to reduce computational complexity. However, this reduction can lead to information loss, especially when the input spectra contain fine-grained details that are crucial for distinguishing subtle variations in temperature. In contrast, feature engineering methods like polynomial approximations and PCA preserve this high-dimensional information more effectively by focusing on extracting relevant features without losing the global context of the spectral data.

Insufficient Feature Representation Learning: CNNs tend to learn hierarchical features through multiple layers of convolutional filters, which is highly beneficial for image data. However, in the case of emission spectra, where the relevant features are not hierarchical in nature, the learned representations may not align with the true underlying patterns. As demonstrated in this study, feature

engineering techniques such as physics-guided transformations and signal representation features provide a more direct representation of the spectral data, allowing classical machine learning models to leverage these features more effectively.

Feature Engineering in Domain-Specific Knowledge: The feature engineering techniques employed in this study incorporate domain-specific knowledge through physics-based transformations and signal representation features. These features are explicitly designed to capture the relationships between temperature, concentration, and spectral intensities. This contrasts with CNNs, which lack such domain-specific priors and rely entirely on the data to learn these relationships. As a result, CNNs often require larger datasets and extensive training to match the performance of models utilizing engineered features."

3.8 I would suggest to add a section that explains why such particular architectures chosen for this regression task. For example, did any architecture outperform others in preliminary tests, or were the choices based on general trends in the field?

Response: Thank you for your insightful comment. As highlighted, very limited work has been done in the field to measure the spatial distribution of temperature, largely due to the inherent complexity of the problem. Most related works primarily relied on Multi-Layer Perceptrons (MLPs) as a solution, but this motivated us to explore a wider range of architectures and methodologies, as there has not been a comprehensive study to determine the most suitable approach for this specific regression task.

In this study, we systematically reviewed and tested various potential solutions, including different feature engineering techniques, classical machine learning models (MLP, SVR, GPR, Random Forest, Blender), and deep learning architectures. The architectural choices were driven by the need to address the complexity of temperature estimation from emission spectra, and we aimed at determining which model could best handle the non-linear and ill-posed nature of this inverse problem.

After extensive experimentation, we found that the "blender light" model consistently outperformed other models across all metrics, including RMSE, RE, RRMSE, and correlation coefficient. This performance guided the final selection of the architecture, confirming that it was the most suitable choice based on empirical evidence from the extensive experiments.

This systematic exploration not only justified the use of the chosen architecture but also filled a gap in the literature by evaluating a broad range of models for this task.

3.9 I would highly suggest the authors to add a comparison of model performance on low-resolution versus high-resolution spectra which could clarify the trade-offs involved in using different spectral resolutions.

Response: Thank you for your valuable suggestion. To support responding to this comment, we would like to clarify why we focused on low-resolution spectra in the present study:

1. **Challenge of Low-Resolution Spectra**: As mentioned in the data generation section, *"low-resolution spectra are affected by blurring, leading to a loss of detail, which increases the complexity of the reconstruction task."* This makes temperature estimation from low-resolution spectra significantly more difficult. For example, methods that perform well on high-resolution spectra, such as the one referenced in [16], do not perform as effectively when applied to the low-resolution data, analyzed in this study.
2. **Practical Considerations**: Low-resolution spectra are easier and more cost-effective to obtain, as they do not require expensive, high-precision spectrometers. This makes them more suitable for widespread engineering applications, where cost and acquisition speed are critical.

Therefore, the focus of the present study was on low-resolution spectra to reflect these practical constraints.

In response to your feedback, we elaborated on this rationale in the revised manuscript as follows:

"The purpose behind this choice is that a lower resolution leads to higher spectral generation speed, and reduces instrument costs, which are often used in engineering applications. In addition, it also increases the degree of complexity of obtaining temperature measurements, thus making the problem even more challenging to solve."

3.10 The research includes only two window lengths, 20 and 50 emission lines. I would suggest that the authors try to incorporate more than just these two window lengths.

Response: Thank you for your valuable suggestion. The selection of two window lengths (20 and 50 emission lines) was made with the intent to balance reducing data dimensionality, while preserving important local patterns, which can be captured by fitting coefficients. As shown in Table 5, a window length of 50 results in a minimal feature set of 270, which is manageable for conventional machine learning algorithms, and still contains sufficient information after this compression. Similarly, a window length of 20 generates around 1,400 features, which, although larger, remains feasible for conventional ML algorithms.

The experimental results demonstrated that these two window lengths yield comparable performance, as reflected in Table 5. Furthermore, the final results presented in Figure 8 and Table 6 demonstrate that these window lengths offer satisfactory performance for the specific research objectives. Therefore, we consider these two window sizes sufficiently representative in the scope of the present study.

That said, we agree with the reviewer that exploring additional window lengths through a more comprehensive hyperparameter search could potentially enhance model performance. We appreciate this suggestion and have incorporated this into future work, as stated in the conclusion:

"More detailed hyper-parameter searches, such as optimizing window size, along with exploring alternative feature engineering methods like other dimensionality reduction techniques, could further enhance performance. Additionally, advanced feature learning techniques, such as contrastive learning and self-supervised learning, may be investigated to extract more effective features."

This statement demonstrates our commitment to further refine and improve the research investigation as part of future work.
