# Supplementary material for: Comparative analysis of data-driven models for spatially resolved thermometry using emission spectroscopy
Source: PLoS One. 2025 Jan 24;20(1):e0317703. doi: 10.1371/journal.pone.0317703 (PMC11760626; doi:10.1371/journal.pone.0317703)
Supplement: Attachment — Submitted filename: PONE-D-24-35923R2- Response to the Reviewers comments.pdf [file pone.0317703.s002.pdf]

Response to the Journal/Academic Editor:

We would like to sincerely thank the reviewers in this second round of review for their insightful comments and constructive suggestions. Their thorough evaluation and thoughtful feedback have greatly contributed to improving the clarity, depth, and overall quality of the submitted manuscript. In the following sections, we provide a point-by-point response to all comments made by the reviewers.

Reviewer #1: The manuscript have been revised and it is more suitable for acceptance to the best of my Knowledge

Thank you very much for your valuable feedback and review.

Reviewer #3: The authors present a very profound research that implements various data-driven models in measuring temperature distributions in a spatially resolved manner using emission spectroscopy data. However, here are few suggestions mentioned below:

1. The related works section has only 5 previous works that have been discussed in this paper. Perhaps the authors should try to add a few more previous related works.

Thank you for your valuable suggestion. We have added more references for emission-spectroscopy-based temperature measurements, and reorganized the section of related work. the new added references are as follows:

5. Parameswaran T, Hughes R, Gogolek P, Hughes P. Gasification temperature measurement with flame emission spectroscopy. Fuel. 2014;134:579–587.

6. Yubero C, García MC, Varo M, Martínez P. Gas temperature determination in microwave discharges at atmospheric pressure by using different Optical Emission Spectroscopy techniques. Spectrochimica Acta Part B: Atomic Spectroscopy. 2013;90:61–67.

17. Park JH, Cho JH, Yoon JS, Song JH. Machine learning prediction of electron density and temperature from optical emission spectroscopy in nitrogen plasma. Coatings. 2021;11(10):1221.

18. Kim D, Bong C, Im Sk, Bak MS. Simultaneous measurement of carbon emission and gas temperature via laser-induced breakdown spectroscopy coupled with machine learning. Optics Express. 2023;31(4):7032–7046.

19. Yi Y, Kun D, Li R, Ni K, Ren W. Accurate temperature prediction with small absorption spectral data enabled by transfer machine learning. Optics Express. 2021;29(25):40699–40709.

2. Add a table that abbreviates all the variables and formulas used in the equations used in the paper so it can be easier for the readers to follow along.

We agree with your recommendation that providing an abbreviation table for all symbols used in the paper would be convenient for readers to follow, however, it is notable that we do not find the available format in the journal template for this function. With this in mind, we provided detailed definitions of the features extracted in the revised Tables 2 and 3.

3. What are the specific reasons to choose such machine learning models for comparison

The selection of these machine learning models was guided by several key considerations, as follows:

Model Diversity: The chosen models represent a diverse range of machine learning approaches, covering major learning paradigms:

- Multi-Layer Perceptron (MLP) as a neural network-based approach
- Gaussian Process Regression (GPR) for probabilistic regression
- Support Vector Regression (SVR) for non-linear regression with kernel methods
- Radial Basis Function Networks (RBFN) for function approximation
- Ensemble methods (Random Forests and Boosted Trees) for providing high prediction robustness and the capability to combine already trained models.

Complementary Strengths:

- MLP can capture complex non-linear relationships through its hidden layers and globally parameterized learning style.

- GPR provides uncertainty quantification and handles small datasets well with non-parameterized learning style
- SVR excels in high-dimensional spaces and manages non-linear relationships
- RBFN is particularly effective for interpolation and function approximation with regional learning style.
- Ensemble methods (Random Forests and Boosted Trees) offer robust predictions by combining multiple weak learners, reducing overfitting and improving generalization

By employing this comprehensive suite of models, we aimed at providing a robust and multi-perspective approach to estimating temperature profiles, ensuring a thorough and systematic investigation of predictive capabilities across different machine learning technologies.

Accordingly, we added the following paragraph to accommodate the reviewer's comment in the manuscript.

"To ensure a robust and comprehensive investigation of temperature profile estimation, a diverse suite of machine learning models was employed, each representing distinct learning paradigms with complementary strengths. The chosen methods include Multi-Layer Perceptron (MLP) [35] for capturing complex non-linear relationships, Gaussian Process Regression (GPR) for its probabilistic predictions and suitability in small datasets [36], Support Vector Regression (SVR) [37] for its efficiency in high-dimensional spaces, and Radial Basis Function Networks (RBFN) [38] for effective interpolation and localized function approximation. Additionally, ensemble methods [39], i.e., Random [40] and Boosted Trees [41] were utilized for their ability to combine multiple weak trees, offering robustness and improved generalization. In addition to the above ML models, a blending method is also employed to provide superior performance based on the aforementioned models' performance through a two-stage training process, as shown in Figure 6. The first stage models are the weak learners, i.e., the ML models used to tackle the problem individually. The second stage of the process is the blending model or meta learner, which provides the final estimates, using the primary estimates from the weak learners. The meta learner can be a linear (e.g., weighted average) or a nonlinear model (e.g., MLP). This diverse model selection ensures that complementary perspectives and capabilities are leveraged, enabling a systematic evaluation of the predictive performance across different machine learning approaches."

In addition to this, we also accommodated a justification for the selection of the specific Deep Learning methodologies in the manuscript, as follows:

"The selection of VGG, Resnet, Inception, Shuffle Net, and Squeeze Net for comparison in this study was guided by their prominence in the field and their architectural diversity. VGG represents one of the earliest and most well-known convolutional neural network architectures, emphasizing deep stacking of layers for feature extraction. Resnet introduces the concept of residual learning through skip connections, which addresses the vanishing gradient problem in deep networks. Inception networks leverage multi-scale convolutional kernels, allowing them to extract features across different receptive fields, making them effective for varied data patterns. Shuffle Net and Squeeze Net, on the other hand, are lightweight architectures designed for computational efficiency, making them practical for scenarios with limited resources. These models were chosen as they collectively cover a wide spectrum

of design philosophies, from classical architectures to modern lightweight designs, ensuring a comprehensive evaluation."

4. Explain the main difference between Convolutional Neural Networks and the machine learning models, perhaps provide a comparison among the results.

In our research, we contrasted the use of Convolutional Neural Networks (CNNs) and traditional machine learning (ML) models according to their difference in feature engineering: classical ML algorithms inherently struggle with high-dimensional data due to the well-documented dimensionality curse. Consequently, feature engineering becomes essential to compressing high-dimensional sparse data into low-dimensional dense features that ML regressors can effectively utilize. CNNs, in contrast, employ convolution filters and gradient backpropagation to learn feature representations automatically, potentially mitigating the dimensionality challenge and reducing manual feature engineering workload. However, it is crucial to note that the CNN's design and convolution filter capabilities may not always guarantee optimal feature representation. In contrast, classical ML regressors offer the flexibility to employ diverse feature extraction and selection methodologies, as highlighted in our response to comment 3, enabling a more nuanced and potentially more accurate feature engineering approach.

Their difference in feature engineering and the corresponding effect on their performance has been discussed in the following narrative in the manuscript:

"Despite the well-documented success of Convolutional Neural Networks in various data-driven applications such as image classification, object detection, and time-series forecasting, their performance in the context of spatially resolved temperature measurement using emission spectroscopy was found to be inferior to the combination of advanced feature engineering and classical machine learning methods in this study. Several key reasons contribute to this outcome:

- *Loss of Spectral Characteristics During Convolution: Spectral data is characterized by its peaks and troughs, which directly correlate with the physical properties of the gas mixture, such as concentration and temperature distribution. Convolutional filters, designed for translation invariance in images, do not inherently capture these spectral characteristics effectively. As a result, important spectral features such as peak intensities and their precise locations might be averaged out during convolution, leading to a loss of critical information necessary for accurate temperature estimation.*
- *Challenges with High-Dimensional Spectral Inputs: The high-dimensional nature of the spectral data (e.g., 6799 emission lines) poses a challenge for CNNs, which rely on down-sampling through pooling layers to reduce computational complexity. However, this reduction can lead to information loss, especially when the input spectra contain fine-grained details that are crucial in distinguishing subtle variations in temperature. In contrast, feature engineering methods, e.g., polynomial approximations and PCA, preserve this high-dimensional information more effectively by focusing on extracting relevant features without losing the global context of the spectral data.*
- *Insufficient Feature Representation Learning: CNNs tend to learn hierarchical features through multiple layers of convolutional filters, which is highly beneficial for image data. However, in the case of emission spectra, where the relevant features are not hierarchical in nature, the learned representations may not align with the true underlying patterns. As demonstrated in this study, feature engineering techniques such as physics-guided transformations and signal representation features provide a more direct representation of the spectral data, allowing classical machine learning models to effectively leverage these features.*

- *Feature Engineering with Domain-Specific Knowledge: The feature engineering techniques employed in this study incorporate domain-specific knowledge through physics-based transformations and signal representation features. They are explicitly designed to capture the relationships between temperature, species concentration, and spectral intensities. This contrasts to CNNs, which lack such domain-specific priors and rely entirely on the data to learn these relationships. As a result, CNN methods often require larger datasets and extensive training to match the performance of models utilizing engineered features."*

5. Make sure to proofread the entire manuscript for any grammatical errors.

Thank you for your recommendation. We would like to confirm that the entire manuscript has been thoroughly proofread and any typographical/grammatical and syntactical errors have been addressed in the revised version.

Reviewer #4: The authors have conducted a greatly in-depth research related to addressing the caveat that line-of-sight emission spectroscopy and made changes accordingly to the first revision comments. Here are few comments I'd like to provide:

1. I'd suggest the authors to provide a small overview for each section and subsection instead of directly jumping between one section to subsection without any overview or introduction. For example, Section 4 not only jumps one subsection but two, it goes from primary section 4 to 4.1, and then directly to 4.1.1.

Thank you for your valuable comment. As recommended by the reviewer, we added the necessary overview to better organize the sections, and improve the flow of the manuscript. The following additions to the manuscript were made:

Section 3:

"...Sec. 3.1 will introduce the formulation of the forward modeling in emission spectroscopy, followed by spectral acquisition. In Sec.3.2, the data-driven methodologies explored in this study will be introduced."

Section 3.1:

"This section primarily covers the data preparation process for data-driven modeling. Sec. 3.1.1 discusses the development of the physical forward model for spectra generation from temperature and mole fraction profiles. In Sec. 3.1.2, the physical model is applied in synthesizing the spectral data for use in the data-driven system development and testing."

Section 4:

"The data-driven methodologies introduced in Sec.3.2 were used in the experimental studies, and the corresponding results are provided below. Sections 4.1 and 4.1 report the performance of the combination of feature engineering and machine learning, and Convolutional Neural Networks, respectively. Sec.4.3 provides a performance comparison between the top performing model in our experiments and the state-of-the-art approaches, demonstrating the effectiveness of the proposed approach. Sources of potential errors and a critical analysis of the limitations of the proposed method are discussed in Sec.4.4."

Section 4.1:

"The high dimensionality and sparsity of the raw spectral data may lead to divergence of ML models, which necessitates the use of feature engineering to extract and select valuable features for information representation. Motivated by the above observation, Sec.4.1.1 demonstrates and discusses the performance of the proposed systematic feature engineering methodology, followed by the presentation and discussion of the optimal feature groups for machine learning modeling, and their corresponding performances in Sec.4.1.2."

2. The conclusion portion of the paper should be presented in a paragraph format to match the appropriate format of a research article

Thank you for your valuable comment, we modified the conclusion section to accommodate the reviewer's comment as follows:

"In this study, the feasibility of using data-driven models to recover spatially resolved temperature distributions from line-of-sight emission spectroscopy data was systematically explored, focusing on two primary approaches, i.e., feature engineering with classical machine learning methods, and end-to-end convolutional neural networks. The research yielded several significant findings. First and foremost, the combination of physics-guided transformation, polynomial approximation-based features, and Principal Component Analysis emerged as the most effective feature extraction methodology. Next, among fifteen traditional machine learning models, the light blender model demonstrated exceptional performance, achieving remarkable metrics including a root mean square error of 64.3, relative error of 0.017, relative root mean square error of 0.025, and an impressive R-value of 0.994. This result not only validates the feasibility of data-driven models in measuring nonuniform spatial temperature distributions but also highlights the substantial advantages of feature engineering.

While end-to-end Convolutional Neural Networks offered competitive performance compared to state-of-the-art techniques, they could not match the performance of feature engineering and machine learning models. This finding underscores the critical role of carefully engineered features in enhancing predictive accuracy for spectroscopic temperature estimation.

The implications of this research extend beyond methodological advancements. The high-performing models of this research have significant potential for improving temperature estimation accuracy across various industrial and research domains. In industrial settings, such as combustion monitoring, materials processing, and chemical reactors, they can enable more precise temperature control, leading to improved operational efficiency, enhanced energy savings, and increased safety. The ability to accurately capture temperature variations across a region opens new possibilities for real-time monitoring systems, potentially reducing manual interventions and facilitating more automated, intelligent measurement techniques.

Looking forward, several promising avenues for future research have been identified. These include exploring the impact of incorporating new data to enhance the performance of both weak learners and meta learner, through retraining and fine-tuning. The objective of this investigation being to optimize the use of the blending approach to leverage larger data populations, potentially leading to improvements in measurement accuracy and reliability. Along a similar direction, we intend to increase the diversity of the datasets to consider broader application scenarios, systematically investigating the impact of hyper-parameter selection, considering advanced feature learning technologies, e.g., contrastive and self-supervised learning, and the use of advanced deep learning architectures and hybrid machine learning approaches, which combine feature engineering with deep learning architectures. Such studies could further refine and extend the capabilities of data-driven temperature estimation techniques, pushing the boundaries of spectroscopic measurement technologies."

3. Are there any specific reasons only the following five CNN models were used for comparison: VGG, Resnet, Inception, Shuffle net, Squeeze net?

Thank you for your insightful comment. We added the following justification for the choice of CNN models in the revised manuscript:

"The selection of VGG, Resnet, Inception, Shuffle Net, and Squeeze Net for comparison in this study was guided by their prominence in the field and their architectural diversity. VGG represents one of the earliest and most well-known convolutional neural network architectures, emphasizing deep stacking of layers for feature extraction. Resnet introduces the concept of residual learning through skip

connections, which addresses the vanishing gradient problem in deep networks. Inception networks leverage multi-scale convolutional kernels, allowing them to extract features across different receptive fields, making them effective for varied data patterns. Shuffle Net and Squeeze Net, on the other hand, are lightweight architectures designed for computational efficiency, making them practical for scenarios with limited resources. These models were chosen as they collectively cover a wide spectrum of design philosophies, from classical architectures to modern lightweight designs, ensuring a comprehensive evaluation."

Nevertheless, the chosen models do not exhaust all potential choices for CNN architectures, however, they provide a substantially representative sample of key paradigms in deep learning for regression and classification tasks, enabling meaningful comparisons with the proposed methods. As mentioned in the Future work section, we intend to expand comparison to include newer architectures or variants optimized for spectral data, providing further insights into the applicability of CNNs for spatially resolved temperature measurements.

4. Have the authors thought about adding another training set in the blending method, and if so, do you believe it can improve the prediction accuracy?

Thank you for your comment. In the blending method, the primary objective is to enhance prediction accuracy by utilizing already trained models (weak learners) to combine their respective strengths. These models are trained on the same dataset, and the meta-learner combines their outputs to improve overall performance. If an additional dataset were to be introduced, it could indeed improve the performance of the weak learners as well as the meta-learner, since the weak learners would gain access to a larger population of data, which typically leads to better generalization and improved accuracy.

When a new dataset is available, the weak learners could be retrained or fine-tuned, allowing them to better capture the underlying patterns in the data. Since the meta-learner works by aggregating the predictions from the weak learners, having stronger individual models due to a richer training set would result in more accurate and reliable predictions from the meta-learner. This would not only improve the blending method's performance but also increase its robustness to different types of data.

As part of our future work, we plan to investigate the effect of additional datasets on the blending method, both in terms of retraining the weak learners and evaluating the overall performance improvements. This could involve experimenting with data augmentation techniques or incorporating domain-specific datasets that enhance the weak learners' ability to generalize across diverse conditions.

To accommodate the reviewer's comment in the revised manuscript, the following sentence was added in the future work part of the Conclusion section:

"*…These include exploring the impact of incorporating new data to enhance the performance of both weak learners and meta learner, through retraining and fine-tuning. The objective of this investigation being to optimize the use of the blending approach to leverage larger data populations, potentially leading to improvements in measurement accuracy and reliability…"*
